# Alteration of prefrontal functional connectivity in preclinical Alzheimer's disease: an fNIRS study

**DOI:** 10.3389/fnagi.2025.1507180

**Published:** 2025-03-11

**Authors:** Minhee Kim, Jang Jae Lee, Kyu Yeong Choi, Byeong C. Kim, Jeonghwan Gwak, Kun Ho Lee, Jae Gwan Kim

**Affiliations:** ^1^Department of Biomedical Science and Engineering, Gwangju Institute of Science and Technology, Gwangju, Republic of Korea; ^2^Gwangju Alzheimer's and Related Dementia Cohort Research Center, Chosun University, Gwangju, Republic of Korea; ^3^Department of Neurology, Chonnam National University Medical School, Gwangju, Republic of Korea; ^4^Department of Software, Korea National University of Transportation, Chungju, Republic of Korea; ^5^Department of Biomedical Science, Chosun University, Gwangju, Republic of Korea; ^6^Korea Brain Research Institute, Daegu, Republic of Korea

**Keywords:** Alzheimer's disease, preclinical stage, functional near-infrared spectroscopy, verbal fluency task, older adult, functional connectivity, prefrontal cortex

## Abstract

**Background:**

Early detection of Alzheimer's disease (AD) is vital for delaying its progression through timely intervention. The preclinical stage, the longest phase of AD, often goes undetected due to a lack of noticeable symptoms. Developing an accessible and quantitative screening method for AD is essential for enabling appropriate interventions during this stage.

**Methods:**

Functional near-infrared spectroscopy was used to investigate prefrontal functional connectivity in preclinical AD subjects. A total of 99 participants, including healthy controls and preclinical subjects who were amyloid beta (Aβ) positive (*n* = 45), were recruited. We designed a mixed phonemic and semantic verbal fluency task for the experimental protocol. Functional connectivity was then analyzed as z-values in the left, right, and interhemispheric prefrontal regions during a verbal fluency task. Finally, we assessed the correlation between the participants' z-values and clinical indices.

**Results:**

The preclinical AD group exhibited increased interhemispheric functional connectivity derived from oxygenated and deoxygenated hemoglobin during verbal tasks involving the first phonemic letter. Additionally, significant right and left functional connectivity differences were observed in the healthy control group during verbal tasks with the letter and categories, but not in the preclinical AD group. Lastly, the difference in interhemispheric functional connectivity of oxygenated hemoglobin between the first and second verbal trials was significantly greater in the preclinical AD group. These interhemispheric functional connectivity values were significantly correlated with Aβ results from positron emission tomography.

**Conclusion:**

The initial increase and subsequent reduction of interhemispheric functional connectivity in the preclinical AD group across task repetitions suggests that task-related prefrontal network alterations may occur during the preclinical phase of AD and shows its potential as a biomarker for screening preclinical AD.

## 1 Introduction

Early detection of Alzheimer's disease (AD) is a crucial factor in delaying the progression of the disease through timely medical intervention. However, the preclinical stage, which is the longest phase of AD, often remains undetected due to the absence of noticeable symptoms (Jack et al., 2010). Furthermore, even when individuals seek early diagnosis, undergoing medical examinations such as positron emission tomography (PET) and magnetic resonance imaging (MRI) scans is both time-consuming and expensive. As a result, people tend not to visit hospitals unless they perceive a significant impairment in their symptoms, which ultimately delays the early detection of AD. Therefore, the development of a readily accessible and quantitative screening method for AD is essential to enable appropriate interventions during the preclinical stage. In this context, functional near-infrared spectroscopy (fNIRS) can be considered a promising alternative.

fNIRS is a promising optical technology that can be widely utilized in clinical research, as it enables the observation of functional changes related to blood oxygenation using relatively compact instruments. Compared to PET and MRI, fNIRS offers significant advantages, such as the absence of radioactive materials and reduced measurement noise due to its reliance on the optical properties of hemoglobin. Furthermore, the probes can be attached to the target area quickly and easily, making fNIRS a practical tool for AD research. In fact, fNIRS has already been applied to investigate hemodynamic impairments associated with dementia caused by AD (Hock et al., 1996). However, further research and validation are required, as the standardization of AD biomarkers using fNIRS remains insufficient.

The biomarker of fNIRS is fundamentally based on changes in hemoglobin concentration in the brain induced by cognitive stimulation. One of the representative brain stimulation tasks is the verbal fluency task (VFT), which is associated with executive function, memory, and language. VFT has also been utilized to detect cognitive impairment (Zhao et al., 2013). The association between VFT and fNIRS has been explored in mild cognitive impairment (MCI) and AD studies, demonstrating its effectiveness. For instance, studies on MCI using VFT have identified several features, including frontal asymmetry alterations (Yeung et al., 2016), reduced brain responses in the frontoparietal region (Katzorke et al., 2018), and decreased prefrontal connectivity (Nguyen et al., 2019). Research on AD dementia has reported reduced brain responses in the frontoparietal region (Metzger et al., 2016) and prefrontal cortex (Herrmann et al., 2008), as well as the loss of frontal lobe asymmetry (Fallgatter et al., 1997). Previous studies using hemoglobin-based biomarkers have primarily focused on groups with progressed clinical symptoms, and their applicability to cognitively normal AD groups has not been investigated. Therefore, we conducted a study to explore fNIRS biomarkers in the preclinical AD group.

This study focused on evaluating differences in functional connectivity (FC) between cognitively healthy individuals and preclinical AD subjects using fNIRS. Specifically, fNIRS was used to examine differences in prefrontal FC during phonemic and semantic VFT in the preclinical stage of AD. FC was calculated using the Pearson correlation coefficient to assess functional synchronization in the right, left, and interhemispheric regions of the prefrontal cortex. Unlike previous studies, this research focused on assessing brain functionality in age- and education-matched healthy individuals (Ho et al., 2022). Furthermore, we investigated whether changes in FC exhibited a linear relationship with Aβ concentration.

## 2 Materials and methods

In this study, we aimed to investigate the difference in the prefrontal FC between healthy elderly and preclinical AD subjects. The study protocol was approved by the Institutional Review Board of Gwangju Institute of Science and Technology (20201124-HR-57-02-04).

### 2.1 Participants

Cognitively healthy individuals aged 65 years or older were recruited from the Gwangju Medical Center for Dementia. Comprehensive cognitive assessments using the Korean Mini-Mental State Examination (K-MMSE) and the Seoul Neuropsychological Screening Battery (SNSB) were conducted to screen individuals without cognitive impairment (Ryu and Yang, 2023). The SNSB evaluates five cognitive domains: attention, language, visuospatial function, memory, and frontal/executive functions for one and a half hours, enabling accurate confirmation of cognitive normality. In this study, which focuses on individuals definitively diagnosed as cognitively normal, the clinical dementia rating was not utilized, as it is primarily designed to assess dementia severity. Subsequently, a three-dimensional structural MRI (MAGNETOM Skyra; Siemens Healthineers, Germany) was performed to detect brain atrophy and severe brain-related diseases, and ^18^F-florbetaben (FBB) PET scans (Discovery STE PET/CT Scanner; GE Healthcare, USA) were conducted to quantify Aβ deposition. Quantification was expressed as the standard uptake value ratio (SUVR), representing Aβ uptake in specific brain regions, including the frontal, precuneus, posterior cingulate cortex, lateral parietal, and temporal regions, normalized by the cerebellar uptake value computed through co-registration with individual T1 postural images. SUVR-A is an automatically calculated SUVR using a deep neural network (Kang et al., 2023). The preclinical stage of AD was defined as the absence of clinical symptoms of atypical AD with *in-vivo* evidence of Alzheimer's pathology, according to the International Working Group (Dubois et al., 2014).

The criteria for group classification were based on whether participants were cognitively normal in cognitive assessments and whether they were classified as amyloid-negative or amyloid-positive in amyloid PET imaging. Based on these criteria, the healthy control (HC) group was categorized as amyloid-negative, and the preclinical AD group was categorized as amyloid-positive. Cognitive health status was determined by combining the K-MMSE and SNSB results, as these tests can quantify the level of cognitive impairment and are widely used for dementia screening in the community. The preclinical AD group did not meet the diagnostic threshold for MCI, which requires scores at least 1.5 SD below the mean in two or more cognitive domains (Jak et al., 2009). Therefore, both groups were considered cognitively normal. Amyloid positivity was determined by medical professionals and PET data were computationally analyzed with SUVR values-typically using a threshold of 1.1-summarized in Table 1 (Fleisher et al., 2013). Participants with missing clinical assessments or data measurement issues were excluded. Additionally, one participant from the HC group was excluded during the analysis due to a discrepancy between low SUVR values and PET positivity.

**Table 1:** Demographics of the participants.

**Demographics**	**Healthy control**	**Preclinical AD**
Number (%)	54 (54.5)	45 (45.5)
Age, years, mean (SD)	72.8 (4.0)	73.5 (3.5)
Female sex, *n* (%)	26 (48.1)	18 (40.0)
Education, years, mean (SD)	11.5 (4.5)	11.7 (4.3)
**Body mass index, kg/m**^2^, ***n*** **(%)**		
≤ 25 (normal)	33 (61.1)	24 (53.3)
> 25 (overweight or obese)	21 (38.9)	21 (46.7)
**Occupation**, ***n*** **(%)**		
White collar	24 (44.4)	24 (53.3)
Blue collar	19 (35.2)	18 (40.0)
Household/Unemployed	11 (20.4)	3 (6.7)
**Household income**, ***n*** **(%)**		
High	3 (5.6)	4 (8.9)
Middle	42 (77.8)	38 (84.4)
Low	9 (16.6)	3 (6.7)
K-MMSE, mean (SD)	28.0 (1.5)	27.8 (1.4)
**Cognitive measures, z-score, mean (SD)**		
SNSB Attention	-0.05 (0.90)	0.08 (1.01)
SNSB Visuospatial	0.4 (0.85)	0.44 (0.55)
SNSB Memory	0.25 (1.01)	0.21 (0.84)
SNSB Frontal	0.35 (0.94)	0.28 (1.0)
**APOE genotype information**		
APOE4 carrier, *n* (%)	20 (37.0)	32 (71.1)
^18^ **F-Florbetaben PET, mean (SD)**		
SUVR	1.01 (0.06)	1.29 (0.15)^†^
SUVR-A	1.21 (0.06)	1.51 (0.16)^†^

^†^p < 10^−21^.

Finally, 54 HCs and 45 preclinical AD participants were included in this study. Detailed information is presented in Table 1.

### 2.2 Experimental paradigm

**Figure 1:**
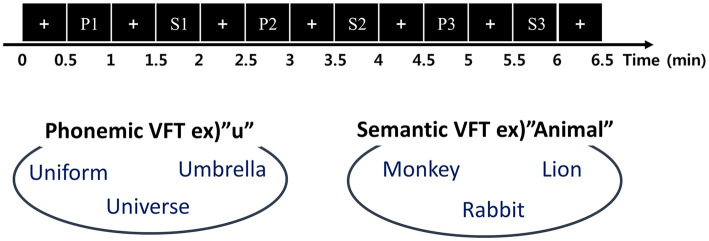
Experimental paradigm of the verbal fluency task.

The VFT included both phonemic and semantic tasks. In the phonemic task, participants were presented with a phonemic cue (e.g., “u”) and instructed to generate words starting with that letter, such as “Uniform” or “Universe.” In the semantic task, participants were given a semantic cue (e.g., “Animal”) and instructed to generate words belonging to that category, like “Lion” or “Cat.” As illustrated in Figure 1, the two conditions were presented alternately to the participants three times for 30 seconds (P1: Korean letter 1, S1: Animal, P2: Korean letter 2, S2: Fruit, P3: Korean letter 3, and S3: Food). The participants were instructed to articulate words continuously until the cue disappeared on the monitor. The interstimulus interval was set to 30 seconds, resulting in a total experimental duration of approximately 330 seconds.

### 2.3 Data aquisition

**Figure 2:**
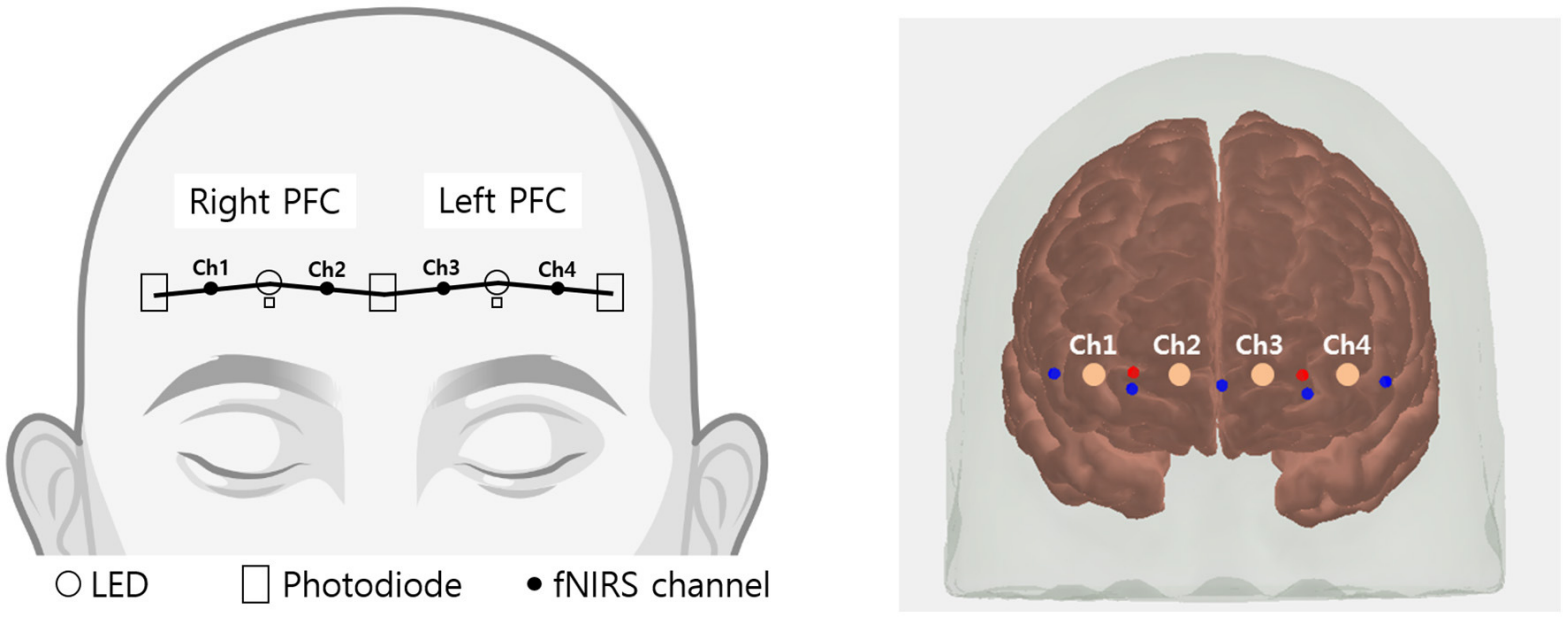
Schematic representation of fNIRS channel locations. Channels 1 and 2 cover the right prefrontal area, while channels 3 and 4 cover the left prefrontal area.

The prefrontal hemoglobin concentration changes were measured using a lab-built fNIRS system. The probe consisted of two LEDs (OE-MV7385-P; OptoENG; Gwangju, Republic of Korea) emitting at wavelengths of 730 and 850 nm, along with five photodiodes (OPT101; Texas Instruments; Dallas, TX, USA). The source-detector separation was 3 cm. The device has been validated for its sensitivity to cognitive stimulation and utilized in a clinical study (Nguyen et al., 2019). fNIRS device consists of two channels on both the right and left sides of the prefrontal regions as shown in Figure 2. Using AtlasViewer, a 3D imaging toolbox based on MATLAB (MathWorks, Natick, MA), projection results indicated that the central photodiode is located near FPz, and two LEDs are located near FP1 and FP2, respectively (Aasted et al., 2015). Accordingly, each fNIRS channel approximately measures the left and right points of FP1 and FP2 (MNI coordinates; channel 1: –42.5, 55.5, –11.7; channel 2: –14.5, 57.0, –2.6; channel 3: 12.6, 58.6, –2.3; channel 4: 38.7, 57.9, –11.0). In addition, the system included two channels with an 8 mm source-detector separation to eliminate systemic physiological changes. After attaching the fNIRS probe, a blackout cloth was placed over the probe to block ambient light. The sampling rate of the fNIRS signal was 8 Hz.

Data preprocessing was conducted using HOMER2 (Huppert et al., 2009), a toolbox designed using MATLAB. Changes in the oxygenated hemoglobin (HbO) and deoxygenated hemoglobin (HbR) concentrations were calculated from the obtained optical density signal using the modified Beer-Lambert law (Delpy et al., 1988). Motion artifacts were identified if a change >8 standard deviations or >0.3 optical density within 0.5 seconds in each channel. The detected motion artifacts were corrected using a cubic spline-correction algorithm (Scholkmann et al., 2010). A low-pass filter with a cutoff frequency of 0.5 Hz was applied to remove cardiac noise (~ 1 Hz) and high-frequency noise. This cutoff frequency is widely used in fNIRS studies as it preserves the overall trend of the fNIRS signal (Jahani et al., 2017; Vermeij et al., 2017). The short-channel data were regressed from the four long-distance channels to enhance the brain hemodynamic response (Yücel et al., 2015). The baseline correction was applied by subtracting mean hemodynamic amplitudes within 20–30 seconds before the first VFT begins for each channel.

### 2.4 Statistical analysis

Statistical analyses were performed using SPSS v29.0.1.0 (IBM Corp., Armonk, NY, USA) and Python v3.7.4. As basic analyses, the mean amplitudes of each VFT trial were compared between the HC and preclinical AD groups. Next, the FC analysis was performed by normalizing the Pearson correlation coefficient between the two preprocessed channels during 60 seconds combined with each VFT trial and following the recovery state using the z-Fisher transformation. Six variables were obtained: one right FC, one left FC, and four interhemispheric FC values. These are referred to as Inter 1 (FC between channels 1 and 3), Inter 2 (FC between channels 1 and 4), Inter 3 (FC between channels 2 and 3), and Inter 4 (FC between channels 2 and 4). To compare demographic variables and fNIRS analysis results between the HC and preclinical AD groups, a two-sided independent *t*-test or Mann-Whitney U test was performed, depending on the data distribution. Effect size Hedges' *g* was computed between groups having different sample sizes and Cohen's *d* was used for analysis within the group. To compare fNIRS channels or FC values within a group, one-way repeated analysis of variance (ANOVA) or Friedman's ANOVA was performed, depending on the data distribution. We applied false discovery rate (FDR) corrections as a post-hoc analysis of multiple observations having pairwise errors of 4 channels and 6 FC values at each VFT trial (Benjamini and Hochberg, 1995). Finally, significant fNIRS outcomes were correlated with clinical biomarkers including SNSB cognitive measures and FBB-PET SUVR values.

## 3 Results

### 3.1 Demographics

The demographic characteristics of the HC and preclinical AD groups are presented in Table 1. To assess the impact of AD on cognitively normal participants, we matched the groups based on age, education, and cognitive measures. The study included 54 HC (mean age = 72.8 years; mean education = 11.5 years; female sex = 48.1 %) and 45 preclinical AD subjects (mean age = 73.5 years; mean education = 11.7 years; female sex = 40.0 %). Amyloid PET analysis revealed a significantly greater SUVR in the preclinical AD group than in the HC group (mean SUVR; HC = 1.01, preclinical AD = 1.29, *p* < 10^−21^; mean SUVR-A; HC = 1.21, preclinical AD = 1.51, *p* < 10^−21^).

### 3.2 Performances during the verbal fluency task

During the phonemic and semantic fluency tasks, both groups generated a similar number of words across trials. The word counts are shown in Table 2. On average, both groups tended to produce more semantic words than phonemic words. There were no significant differences in the number of words produced between the groups.

**Table 2:** The number of words produced during verbal fluency task.

**VFT**	**HC**	**Preclinical AD**	***p*-value**
P1	6.6 (3.0)	6.8 (2.9)	0.751
S1	9.4 (2.7)	9.5 (2.4)	0.904
P2	6.8 (2.7)	6.9 (2.8)	0.893
S2	7.3 (1.9)	7.4 (2.4)	0.730
P3	6.3 (2.6)	6.2 (2.0)	0.799
S3	7.0 (2.2)	7.3 (2.3)	0.589

### 3.3 Concentration change

**Figure 3:**
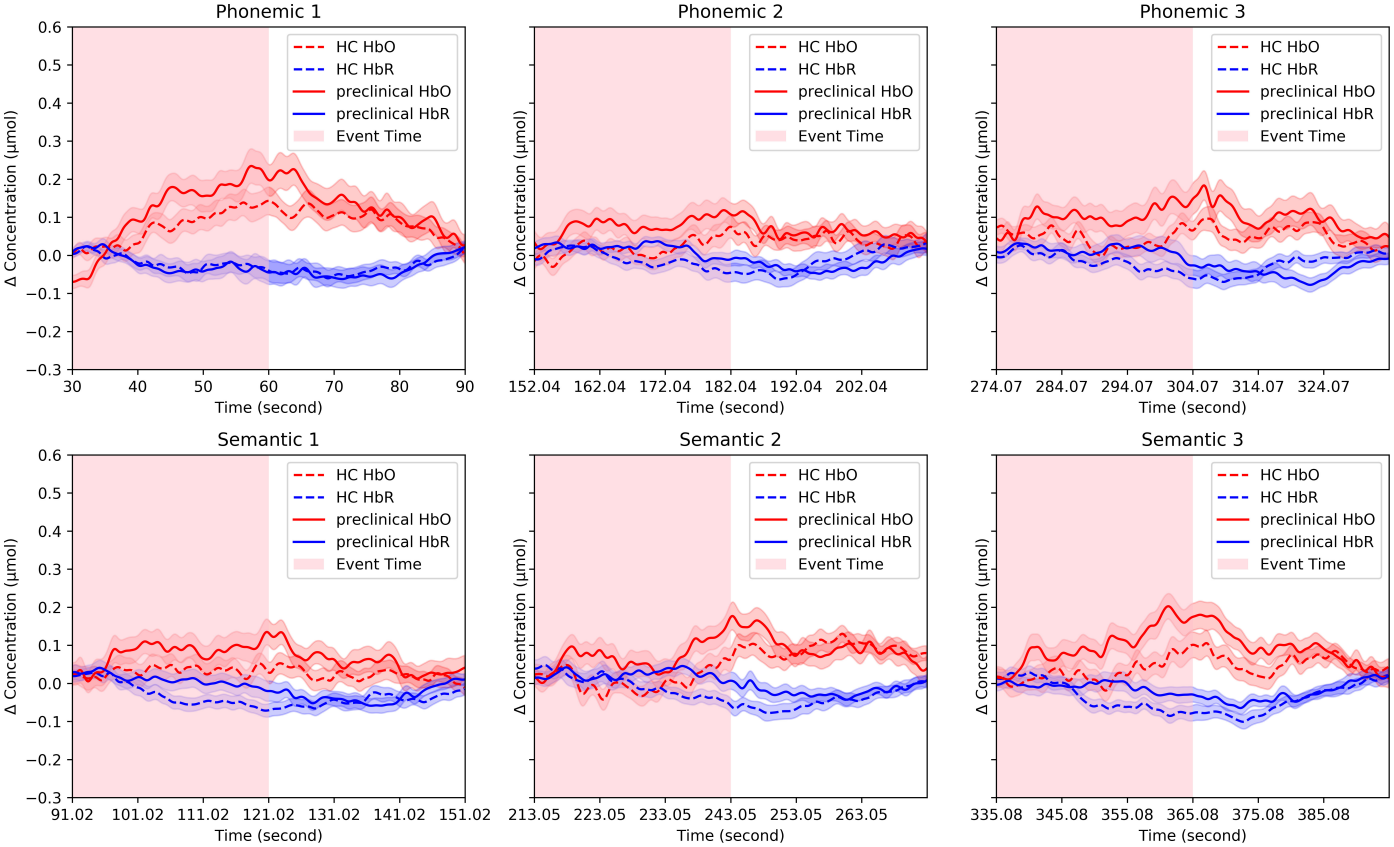
Representative fNIRS signals. Changes in oxygenated and deoxygenated hemoglobin concentrations were averaged for channels and each group. The solid line indicates the preclinical group, and the dotted line indicates the HC group and the pink and blue shaded areas surrounding the solid and dotted lines represent the standard errors.

The group-averaged HbO and HbR signals are presented in Figure 3 for the HC and preclinical AD groups. Changes in HbO and HbR were averaged for channels and each group. In the phonemic and semantic VFTs of grand-averaged data, the preclinical AD group showed greater mean HbO levels than the HC group during P2 (HC = 0.017 ± 0.018 μmol; preclinical AD = 0.077 ± 0.023 μmol; *t*(97) = –2.0; *p* < 0.05; *g* = –0.41), P3 (HC = 0.044 ± 0.017 μmol; preclinical AD = 0.101 ± 0.029 μmol; *p* < 0.05; *g* = –0.35), and S3 (HC = 0.029 ± 0.022 μmol; preclinical AD = 0.100 ± 0.026 μmol; *t*(97) = –2.1; *p* < 0.05; *g* = –0.43; Mean ± Standard deviation). However, no significant differences were found between the two groups in any of the four-channel comparisons with FDR correction.

### 3.4 Prefrontal functional connectivity

#### 3.4.1 Group analysis

**Figure 4:**
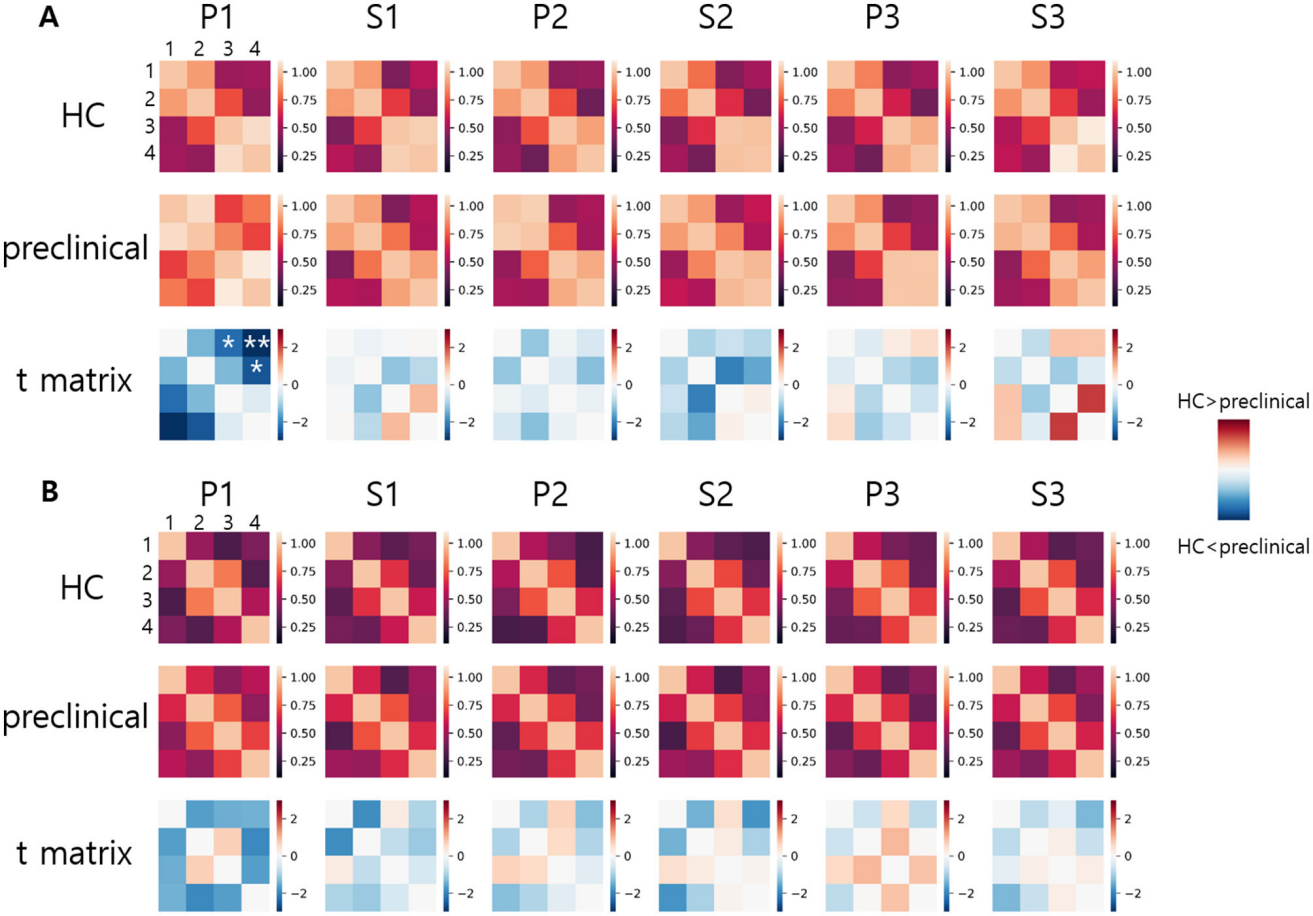
Functional connectivity graph: **(A)** Oxygenated hemoglobin, **(B)** Deoxygenated hemoglobin. Functional connectivity values are displayed in a 4 × 4 matrix. Numbers 1 to 4 indicate the channel numbers. Asterisks indicate statistically significant differences between the healthy control and preclinical groups (FDR corrected, *p < 0.05 and **p < 0.01).

**Figure 5:**
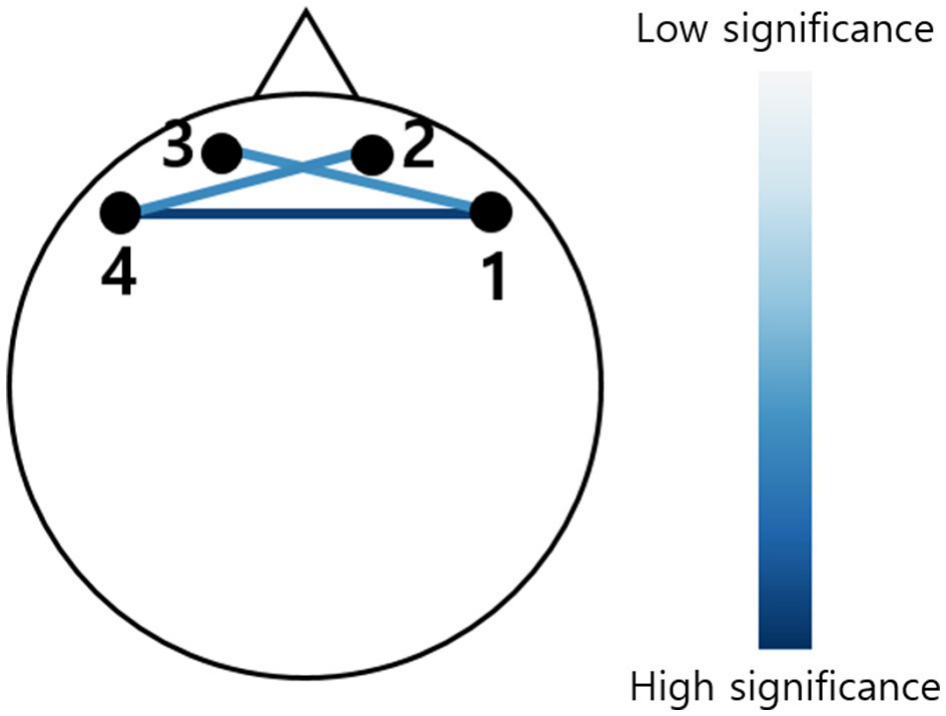
Functional connectivity graph derived from the change in oxygenated hemoglobin. The color indicates the *t*-score, showing only statistically significant connectivity during the first phonemic verbal fluency task (P1) (FDR corrected, *p* < 0.05). The darker blue indicates greater significance between the HC and preclinical AD groups.

The FC from HbO and HbR during each VFT is illustrated in Figures 4A, B. Compared to the HC group, the preclinical AD group showed greater interhemispheric FC of HbO during P1 (Inter 1: HC = 0.49 ± 0.40; preclinical AD = 0.70 ± 0.49; *t*(97) = –2.3; FDR corrected *p* < 0.05; *g* = –0.46; Inter 2: HC = 0.50 ± 0.41; preclinical AD = 0.82 ± 0.45; *t*(97) = –3.6; FDR corrected *p* < 0.01; *g* = –0.73; Inter 4: HC = 0.47 ± 0.44; preclinical AD = 0.71 ± 0.48; *t*(97) = –2.6; FDR corrected *p* < 0.05; *g* = -0.51; Mean ± Standard deviation). The FC graph is presented in Figure 5, showing that the interhemispheric FC from HbO was significantly higher, particularly during P1. In contrast, FC from HbR showed no significance between the groups.

To compare the FC between the right and left prefrontal cortex, the group averaged z-scores are summarized in Table 3 for HbO and Table 4 for HbR. The left FC from HbO had a significantly greater z-score in the HC group during P1, S2, and S3 (P1: *t*(53) = –2.2, *p* < 0.05; *d* = –0.30; S2: *t*(53) = –2.4, *p* < 0.05; *d* = –0.33; S3: *t*(53) = –3.4, *p* < 0.01; *d* = –0.46). For HbR, significant right-left FC difference was observed during S2 in the HC group (*t*(53) = –2.5; *p* < 0.05; *d* = –0.34). Conversely, the preclinical AD group showed no significances observed for HbO and HbR.

**Table 3:** Right and left prefrontal functional connectivity derived from the oxygenated hemoglobin during the verbal fluency task.

	**HC**		**Preclinical AD**	
**VFT z-score, mean (SD)**	**Right PFC**	**Left PFC**	**Right PFC**	**Left PFC**
P1	0.90 (0.52)	1.06 (0.58)^*^	1.06 (0.64)	1.11 (0.62)
S1	0.89 (0.45)	1.03 (0.60)	0.91 (0.45)	0.91 (0.60)
P2	0.90 (0.57)	0.91 (0.56)	1.02 (0.48)	0.93 (0.56)
S2	0.80 (0.50)	0.99 (0.61)^*^	0.90 (0.53)	0.97 (0.48)
P3	0.84 (0.48)	0.94 (0.49)	0.87 (0.47)	1.00 (0.47)
S3	0.88 (0.57)	1.11 (0.46)^**^	0.96 (0.44)	0.91 (0.50)

^*^p < 0.05, ^**^p < 0.01.

**Table 4:** Right and left prefrontal functional connectivity derived from deoxygenated hemoglobin during the verbal fluency task.

	**HC**		**Preclinical AD**	
**VFT z-score, mean (SD)**	**Right PFC**	**Left PFC**	**Right PFC**	**Left PFC**
P1	0.48 (0.48)	0.54 (0.48)	0.63 (0.42)	0.70 (0.50)
S1	0.45 (0.44)	0.60 (0.53)	0.62 (0.51)	0.65 (0.50)
P2	0.53 (0.55)	0.64 (0.46)	0.63 (0.51)	0.67 (0.53)
S2	0.44 (0.61)	0.67 (0.46)^*^	0.61 (0.54)	0.66 (0.51)
P3	0.57 (0.57)	0.70 (0.51)	0.64 (0.52)	0.61 (0.53)
S3	0.52 (0.51)	0.65 (0.50)	0.59 (0.40)	0.62 (0.51)

^*^p < 0.05.

**Figure 6:**
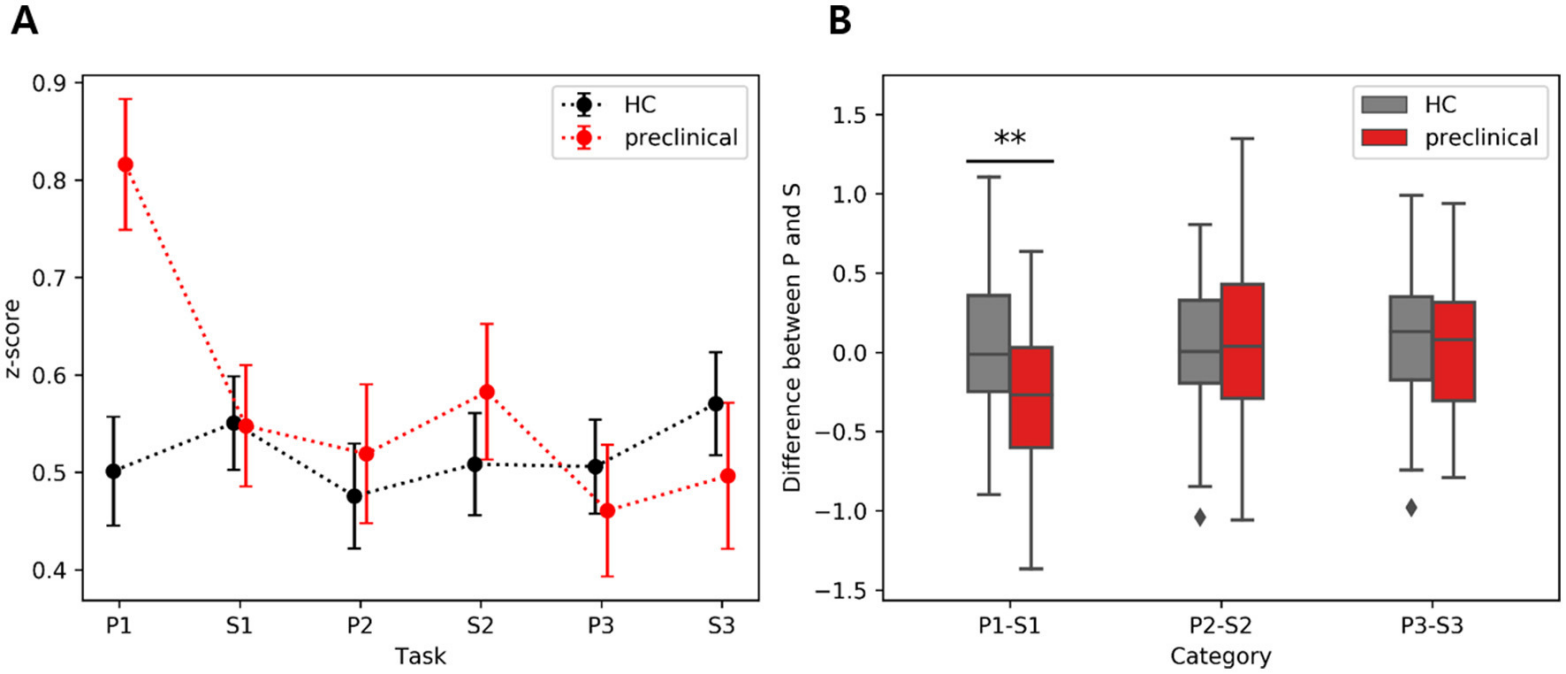
Interhemispheric functional connectivity difference between phonemic (P) and semantic (S) verbal fluency tasks derived from HbO: **(A)** Average of inter2 FC during VFT, **(B)** Inter FC difference between phonemic and semantic VFT. The scatter plots on the left show the mean and standard error, while the boxplots on the right display the interquartile range, with whiskers extending to the lower and upper bound. The asterisk indicates FDR corrected *p* < 0.01.

To investigate the reduction in FC during the transition between P1 and S1, we computed the differences in 6 z-scores between P1 and S1 within each group and also between the two groups. In the inter-group comparison, the preclinical AD group exhibited a significant reduction in interhemispheric FC from HbO (Inter 1: *t*(44) = 3.8; FDR corrected *p* < 0.01; *d* = 0.57; Inter 2: *t*(44) = 3.6; FDR corrected *p* < 0.01; *d* = 0.54), whereas the HC group showed no significant changes. Within-group comparisons revealed no significant differences between P2 and S2, P3 and S3, or FC from HbR. We further compared the extent of FC reduction in HbO between the two groups. The difference in Inter 2 FC during P1 to S1 demonstrated the largest group difference (*t*(97) = 3.3, FDR corrected *p* < 0.01; *g* = 0.67), as illustrated in Figure 6. Additionally, the difference in left FC during P3 to S3 showed a significance (*t*(97) = 2.7, FDR corrected *p* < 0.05; *g* = 0.55). Detailed results for right, left, and other interhemispheric connectivity in both HbO and HbR are provided in the Supplementary material.

#### 3.4.2 Correlation of FC with clinical markers

Since these markers showed the most significance, the interhemispheric FC in P1 and the difference between P1 and S1 were chosen as markers to determine the correlation between SUVR from PET. For comparison with the FC results, the correlation between the average HbO amplitude by channel and clinical variables was examined. The results indicated a very low correlation in S3 (*r* (SNSB Frontal) = 0.23, *p* < 0.05; *r* (SUVR) = 0.24, *p* < 0.05; *r* (SUVR-A) = 0.22, *p* < 0.05). In addition, the average z-scores for the four interhemispheric FCs were computed. As shown in Table 5, positive correlations for interhemispheric FC during P1 were found (Inter 1: *r* (SUVR-A) = 0.21; FDR corrected *p* < 0.05; Inter 2: *r* (SUVR) = 0.28; FDR corrected *p* < 0.05; *r* (SUVR-A) = 0.29; FDR corrected *p* < 0.05; Inter 4: *r* (SUVR-A) = 0.25, FDR corrected *p* < 0.05), and negative correlations were observed in the difference between P1 and S1 (Inter 1: *r* (SUVR) = − 0.23; FDR corrected *p* < 0.05; *r* (SUVR-A) = − 0.23; FDR corrected *p* < 0.05; Inter 2: *r* (SUVR) = − 0.34; FDR corrected *p* < 0.01; *r* (SUVR-A) = − 0.30; FDR corrected *p* < 0.05). Inter 2 had the most significant correlation with both P1 and the difference between P1 and S1 with PET biomarkers.

**Table 5:** Correlation between interhemispheric functional connectivity values and demographic variables.

	**Age**	**Education**	**K-MMSE**	**SNSB attention**	**SNSB language**	**SNSB visuospatial**	**SNSB memory**	**SNSB frontal**	**SUVR**	**SUVR-A**
**Amplitude of channel averaged HbO**										
P2	–0.01	0.10	0.12	–0.02	–0.07	0.11	–0.03	0.12	0.15	0.15
S2	0.13	0.02	–0.01	0.04	–0.15	–0.03	–0.07	0.15	0.17	0.13
S3	–0.04	–0.02	–0.02	0.00	–0.02	0.10	0.07	0.23^*^	0.24^*^	0.22^*^
**FC during P1**										
Inter 1	0.15	–0.04	–0.06	–0.15	–0.10	–0.07	0.00	–0.01	0.16	0.21^*^
Inter 2	–0.04	0.00	–0.01	–0.03	–0.09	0.06	0.04	0.07	0.28^*^	0.29^*^
Inter 3	0.15	0.09	0.10	–0.03	–0.04	–0.05	0.06	0.02	0.09	0.15
Inter 4	–0.08	0.10	–0.01	0.02	–0.01	0.01	0.13	0.07	0.19	0.25^*^
**FC Difference (P1, S1)**										
Inter 1	–0.22	0.05	–0.14	0.08	0.00	–0.08	–0.04	–0.09	–0.23^*^	–0.23^*^
Inter 2	–0.11	–0.01	–0.08	0.06	–0.11	–0.06	–0.07	–0.12	–0.34^**^	–0.30^*^
Inter 3	–0.04	–0.08	–0.18	–0.05	–0.17	–0.19	–0.22	–0.24	–0.07	–0.05
Inter 4	0.02	–0.06	0.03	0.05	–0.18	–0.13	–0.20	–0.12	–0.16	–0.20

^*^FDR corrected *p* < 0.05, ^**^FDR corrected *p* < 0.01.

## 4 Discussion

This study presents a distinctive examination of prefrontal hemodynamics and FC in preclinical AD using a trial-based VFT analysis. The preclinical AD group showed increased interhemispheric FC during the first phonemic VFT and reduced interhemispheric FC during subsequent semantic VFT. Additionally, the preclinical AD group did not exhibit right-left FC differences in prefrontal cortex across the six VFT trials. Lastly, the interhemispheric FC features showed significant correlations with clinical variables, particularly with the PET results.

The absence of difference between right and left FC in preclinical AD may indicate early compensatory mechanisms or diffuse activation patterns linked to AD. However, HbO concentration did not exhibit a significant difference among fNIRS channels, which conflicts with the findings from FC. This discrepancy can be interpreted as a difference in the meanings of the two indices. Hemispheric activation derived from HbO concentration reflects the activity of local neurons near each channel, whereas FC, although coupled with local neural activity, represents activation and inhibition associated with cognitive workload (Tsurugizawa et al., 2023).

For prefrontal cortex, differences in right-left FC were not observed in patients with mild AD, whereas significant left FC was noted in HC (Chan et al., 2020). Similarly, prior studies on prefrontal FC in MCI patients suggested reduced inter- and left-hemispheric FC compared with cognitively healthy individuals (Nguyen et al., 2019). Thus, changes in prefrontal hemodynamics and FC may demonstrate their utility in understanding neurodegenerative diseases, as evidenced by the loss of prefrontal right-left FC differences and increased interhemispheric connectivity observed in preclinical AD in this study.

Our results highlighted that FC changes were more pronounced in HbO than in HbR signals, reflecting the stronger association of HbO with cognitive tasks. This discrepancy may stem from differences in reactivity between HbO and HbR. Importantly, the significant FC changes observed in HbO were partially correlated with PET findings, suggesting a relationship between interhemispheric connectivity and Aβ deposition. While most of the correlation values were very low (~ 0.2), values above 0.3 can be considered meaningful (Ratner, 2009), supporting the interpretation that Inter 2 FC is partially related to amyloid pathology.

In this study, we investigated the brain functions underlying the signal changes observed in preclinical AD during VFT performance. Previous studies utilizing VFT have demonstrated its potential as a biomarker for dementia caused by AD (Henry et al., 2004). VFT is closely associated with executive function, which plays a crucial role in clustering and switching during word generation within specific categories (Shao et al., 2014). Executive function involves the frontal and temporal regions, and VFT is commonly used to study prefrontal brain responses in AD. Impaired executive function has been observed in both MCI and AD (Murphy et al., 2006). This suggests that the findings of our study may be related to executive functioning in preclinical AD. Indeed, evidence indicating a decline in executive performance prior to memory impairment in preclinical AD with Aβ biomarkers supports our results.

Our exploratory findings focus on the prefrontal area, which may limit interpretation in terms of the global hemispheric effect. However, our results enable us to demonstrate prefrontal connectivity in the preclinical phase using a compact system. The PET results exhibited a significant correlation with the interhemispheric FC from fNIRS prefrontal measurements, suggesting that Aβ deposition may impact interhemispheric prefrontal functionality even in the preclinical stage. The preclinical phase represents an initial stage characterized by the accumulation of neurofibrillary tangles (NFTs) in the entorhinal cortex and the deposition of Aβ in the neocortex (Thal et al., 2002). Notably, Aβ is the first detectable biomarker preceding NFT (Jack et al., 2010).

Alteration of default mode network FC in the preclinical AD stage has been observed by using fMRI, with decreased FC in the ventral medial prefrontal cortex and increased FC in the dorsal medial prefrontal cortex, indicating that Aβ modulates prefrontal functional connectivity (Mormino et al., 2011). In the dementia stage of AD, major hemispheric networks exhibit impaired FC, while individuals with MCI demonstrate enhanced activation in the precuneus, suggesting a compensatory effect (Liao et al., 2018). Thus, the observed increase in prefrontal interhemispheric FC in preclinical AD may be associated with a compensatory mechanism that engages cognitive reserve in the early pathological stages of AD (Sperling et al., 2011). Furthermore, our single-trial analysis revealed enhanced activation, particularly during the initial VFT, and subsequent deactivation from the next VFT trial, indicating the initial engagement of interhemispheric function in preclinical AD.

Despite the distinct findings observed in preclinical AD, several confounding factors exist in the experimental methodology. The first factor is the task adaptation effect. All participants underwent a task adaptation phase before the experiment to ensure equal understanding of the task rules. This standardized adaptation process mitigated potential confounds, as evidenced by the absence of group differences in task performance. However, significant differences in FC were observed in the preclinical AD group, indicating that greater interhemispheric interaction is required to initiate cognitive stimulation in preclinical AD.

The second factor is the task order effect. Since the experimental tasks were conducted in a fixed order, a methodological constraint remains regarding whether the hyperactivation observed during the first-trial effect is specifically associated with certain types of VFT, such as phonemic or semantic tasks. Therefore, further study should implement a randomized order of trial to analyze order effects and determine the impact of phonemic and semantic VFTs in preclinical AD.

Moreover, our criteria for diagnosing AD are based on the accumulation level of Aβ within the brain. Demographic analysis revealed that over 70% individuals with preclinical AD carry the APOE4 allele, a known risk factor for AD (Corder et al., 1993). Therefore, increased FC may be partially associated with this genetic risk factor. Consistent with this finding, individuals with a high-risk APOE4 allele exhibit greater activation in the right inferior frontal junction and less activation in the left middle frontal gyrus than those without risk factors (Katzorke et al., 2017).

Utilizing a compact lab-built fNIRS system, this exploratory study demonstrated increased interhemispheric FC during the first phonemic VFT in preclinical AD. While the limited number of channels may restrict detailed analysis of global FC changes, the observed loss of right-left FC differences and significant FC changes provide valuable insights into early AD pathology. Future research should investigate whether the FC changes observed in this study can predict cognitive decline over time. Additionally, integrating fNIRS with other imaging modalities such as PET or MRI could enhance its diagnostic utility and provide a more comprehensive understanding of preclinical AD.

## 5 Conclusion

Identification of the prefrontal functionality of AD is challenging due to limited information. However, with progressive validation utilizing current imaging modalities, minimal and easy techniques for AD screening could guide population-based data collection in the future. Our study investigated FC between the channels of the prefrontal region using hemodynamic changes during a cognitive task in healthy older and preclinical AD individuals. With a cognitive stimulator, which we utilized in the VFT, we observed a substantial increase in interhemispheric FC at the onset of the phonemic VFT and a significant decrease in the subsequent semantic VFT in the preclinical group. These observations, which also correlated with PET biomarkers, suggest that prefrontal FC can be indicative of preclinical functionality for AD. In future studies, exploring whether these outcomes are associated with AD's multiple neurodegenerative stages would be valuable.

## Data Availability

The raw data supporting the conclusions of this article will be made available by the corresponding authors on reasonable request.

## References

[B1] Aasted C. M. Yücel M. A. Cooper R. J. Dubb J. Tsuzuki D. Becerra L.. (2015). Anatomical guidance for functional near-infrared spectroscopy: atlasviewer tutorial. Neurophotonics 2, 020801–020801. 10.1117/1.NPh.2.2.020801 26157991 PMC4478785

[B2] Benjamini Y. Hochberg Y. (1995). Controlling the false discovery rate: a practical and powerful approach to multiple testing. J. R. Stat. Soc. 57, 289–300. 10.1111/j.2517-6161.1995.tb02031.x

[B3] Chan Y. L. Ung W. C. Lim L. G. Lu C.-K. Kiguchi M. Tang T. B. (2020). Automated thresholding method for fnirs-based functional connectivity analysis: validation with a case study on Alzheimer's disease. IEEE Trans. Neural Syst. Rehabil. Eng. 28, 1691–1701. 10.1109/TNSRE.2020.3007589 32746314

[B4] Corder E. H. Saunders A. M. Strittmatter W. J. Schmechel D. E. Gaskell P. C. Small G.. (1993). Gene dose of apolipoprotein e type 4 allele and the risk of Alzheimer's disease in late onset families. Science 261, 921–923. 10.1126/science.8346443 8346443

[B5] Delpy D. T. Cope M. van der Zee P. Arridge S. Wray S. Wyatt J. (1988). Estimation of optical pathlength through tissue from direct time of flight measurement. Phys. Med. Biol. 33:1433. 10.1088/0031-9155/33/12/008 3237772

[B6] Dubois B. Feldman H. H. Jacova C. Hampel H. Molinuevo J. L. Blennow K.. (2014). Advancing research diagnostic criteria for Alzheimer's disease: the IWG-2 criteria. Lancet Neurol. 13, 614–629. 10.1016/S1474-4422(14)70090-0 24849862

[B7] Fallgatter A. Roesler M. Sitzmann A. Heidrich A. Mueller T. Strik W. (1997). Loss of functional hemispheric asymmetry in Alzheimer's dementia assessed with near-infrared spectroscopy. Cogn. Brain Res. 6, 67–72. 10.1016/S0926-6410(97)00016-5 9395850

[B8] Fleisher A. S. Chen K. Liu X. Ayutyanont N. Roontiva A. Thiyyagura P.. (2013). Apolipoprotein e ε4 and age effects on florbetapir positron emission tomography in healthy aging and Alzheimer disease. Neurobiol. Aging 34, 1–12. 10.1016/j.neurobiolaging.2012.04.017 22633529

[B9] Henry J. D. Crawford J. R. Phillips L. H. (2004). Verbal fluency performance in dementia of the Alzheimer's type: a meta-analysis. Neuropsychologia 42, 1212–1222. 10.1016/j.neuropsychologia.2004.02.001 15178173

[B10] Herrmann M. J. Langer J. B. Jacob C. Ehlis A.-C. Fallgatter A. J. (2008). Reduced prefrontal oxygenation in Alzheimer disease during verbal fluency tasks. Am. J. Geriat. Psychiat. 16, 125–135. 10.1097/JGP.0b013e3180cc1fbc 17998307

[B11] Ho T. K. K. Kim M. Jeon Y. Kim B. C. Kim J. G. Lee K. H.. (2022). Deep learning-based multilevel classification of Alzheimer's disease using non-invasive functional near-infrared spectroscopy. Front. Aging Neurosci. 14:810125. 10.3389/fnagi.2022.810125 35557842 PMC9087351

[B12] Hock C. Villringer K. Müller-Spahn F. Hofmann M. Schuh-Hofer S. Heekeren H.. (1996). Near infrared spectroscopy in the diagnosis of Alzheimer's disease a. Ann. N. Y. Acad. Sci. 777, 22–29. 10.1111/j.1749-6632.1996.tb34397.x 8624087

[B13] Huppert T. J. Diamond S. G. Franceschini M. A. Boas D. A. (2009). Homer: a review of time-series analysis methods for near-infrared spectroscopy of the brain. Appl. Opt. 48, D280–D298. 10.1364/AO.48.00D280 19340120 PMC2761652

[B14] Jack C. R. Knopman D. S. Jagust W. J. Shaw L. M. Aisen P. S. Weiner M. W.. (2010). Hypothetical model of dynamic biomarkers of the Alzheimer's pathological cascade. Lancet Neurol. 9, 119–128. 10.1016/S1474-4422(09)70299-6 20083042 PMC2819840

[B15] Jahani S. Fantana A. L. Harper D. Ellison J. M. Boas D. A. Forester B. P.. (2017). fnirs can robustly measure brain activity during memory encoding and retrieval in healthy subjects. Sci. Rep. 7:9533. 10.1038/s41598-017-09868-w 28842618 PMC5572719

[B16] Jak A. J. Bondi M. W. Delano-Wood L. Wierenga C. Corey-Bloom J. Salmon D. P.. (2009). Quantification of five neuropsychological approaches to defining mild cognitive impairment. Am. J. Geriatr. Psychiat. 17, 368–375. 10.1097/JGP.0b013e31819431d5 19390294 PMC2743175

[B17] Kang S. K. Kim D. Shin S. A. Kim Y. K. Choi H. Lee J. S. (2023). Fast and accurate amyloid brain pet quantification without MRI using deep neural networks. J. Nuclear Med. 64, 659–666. 10.2967/jnumed.122.264414 36328490 PMC10071781

[B18] Katzorke A. Zeller J. B. Müller L. D. Lauer M. Polak T. Deckert J.. (2018). Decreased hemodynamic response in inferior frontotemporal regions in elderly with mild cognitive impairment. Psychiat. Res. Neuroimag. 274, 11–18. 10.1016/j.pscychresns.2018.02.003 29472145

[B19] Katzorke A. Zeller J. B. Müller L. D. Lauer M. Polak T. Reif A.. (2017). Reduced activity in the right inferior frontal gyrus in elderly APOE-E4 carriers during a verbal fluency task. Front. Hum. Neurosci. 11:46. 10.3389/fnhum.2017.00046 28220068 PMC5292419

[B20] Liao Z. L. Tan Y. F. Qiu Y. J. Zhu J. P. Chen Y. Lin S. S.. (2018). Interhemispheric functional connectivity for Alzheimer's disease and amnestic mild cognitive impairment based on the triple network model. J. Zhejiang Univ. Sci. B 19:924. 10.1631/jzus.B1800381 30507076 PMC6305256

[B21] Metzger F. G. Schopp B. Haeussinger F. B. Dehnen K. Synofzik M. Fallgatter A. J.. (2016). Brain activation in frontotemporal and Alzheimer's dementia: a functional near-infrared spectroscopy study. Alzheimer's Res. Ther. 8, 1–12. 10.1186/s13195-016-0224-8 27931245 PMC5146884

[B22] Mormino E. C. Smiljic A. Hayenga A. O. H Onami S. Greicius M. D.. (2011). Relationships between beta-amyloid and functional connectivity in different components of the default mode network in aging. Cerebral cortex 21, 2399–2407. 10.1093/cercor/bhr025 21383234 PMC3169663

[B23] Murphy K. J. Rich J. B. Troyer A. K. (2006). Verbal fluency patterns in amnestic mild cognitive impairment are characteristic of Alzheimer's type dementia. J. Int. Neuropsychol. Soc. 12, 570–574. 10.1017/S1355617706060590 16981610

[B24] Nguyen T. Kim M. Gwak J. Lee J. J. Choi K. Y. Lee K. H.. (2019). Investigation of brain functional connectivity in patients with mild cognitive impairment: a functional near-infrared spectroscopy (fNIRS) study. J. Biophot. 12:e201800298. 10.1002/jbio.201800298 30963713

[B25] Ratner B. (2009). The correlation coefficient: Its values range between +1/−1, or do they? J. Target Meas Anal. Mark 17, 139–142. 10.1057/jt.2009.5

[B26] Ryu H. J. Yang D. W. (2023). The Seoul neuropsychological screening battery (SNSB) for comprehensive neuropsychological assessment. Dem. Neurocogn. Disor. 22:1. 10.12779/dnd.2023.22.1.1 36814700 PMC9939572

[B27] Scholkmann F. Spichtig S. Muehlemann T. Wolf M. (2010). How to detect and reduce movement artifacts in near-infrared imaging using moving standard deviation and spline interpolation. Physiol. Meas. 31:649. 10.1088/0967-3334/31/5/004 20308772

[B28] Shao Z. Janse E. Visser K. Meyer A. S. (2014). What do verbal fluency tasks measure? Predictors of verbal fluency performance in older adults. Front. Psychol. 5:772. 10.3389/fpsyg.2014.00772 25101034 PMC4106453

[B29] Sperling R. A. Aisen P. S. Beckett L. A. Bennett D. A. Craft S. Fagan A. M.. (2011). Toward defining the preclinical stages of Alzheimer's disease: recommendations from the national institute on aging-Alzheimer's association workgroups on diagnostic guidelines for Alzheimer's disease. Alzheimer's Dement. 7, 280–292. 10.1016/j.jalz.2011.03.003 21514248 PMC3220946

[B30] Thal D. R. Rüb U. Orantes M. Braak H. (2002). Phases of aβ-deposition in the human brain and its relevance for the development of AD. Neurology 58, 1791–1800. 10.1212/WNL.58.12.1791 12084879

[B31] Tsurugizawa T. Taki A. Zalesky A. Kasahara K. (2023). Increased interhemispheric functional connectivity during non-dominant hand movement in right-handed subjects. Iscience 26:107592. 10.1016/j.isci.2023.107592 37705959 PMC10495657

[B32] Vermeij A. Kessels R. P. Heskamp L. Simons E. M. Dautzenberg P. L. Claassen J. A. (2017). Prefrontal activation may predict working-memory training gain in normal aging and mild cognitive impairment. Brain Imaging Behav. 11, 141–154. 10.1007/s11682-016-9508-7 26843001 PMC5415588

[B33] Yeung M. K. Sze S. L. Woo J. Kwok T. Shum D. H. Yu R.. (2016). Altered frontal lateralization underlies the category fluency deficits in older adults with mild cognitive impairment: a near-infrared spectroscopy study. Front. Aging Neurosci. 8:59. 10.3389/fnagi.2016.00059 27065857 PMC4809883

[B34] Yücel M. A. Selb J. Aasted C. M. Petkov M. P. Becerra L. Borsook D.. (2015). Short separation regression improves statistical significance and better localizes the hemodynamic response obtained by near-infrared spectroscopy for tasks with differing autonomic responses. Neurophotonics 2, 035005–035005. 10.1117/1.NPh.2.3.035005 26835480 PMC4717232

[B35] Zhao Q. Guo Q. Hong Z. (2013). Clustering and switching during a semantic verbal fluency test contribute to differential diagnosis of cognitive impairment. Neurosci. Bull. 29, 75–82. 10.1007/s12264-013-1301-7 23322003 PMC5561862

