# Supplementary material for: Alteration of prefrontal functional connectivity in preclinical Alzheimer's disease: an fNIRS study
Source: Front Aging Neurosci. 2025 Mar 11;17:1507180. doi: 10.3389/fnagi.2025.1507180 (PMC11933025; doi:10.3389/fnagi.2025.1507180)
Supplement: Supplementary file 1 [file Data_Sheet_1.pdf]

Alteration of prefrontal functional connectivity in preclinical Alzheimer's disease : an fNIRS study

Supplymentary material

1. Functional connectivity derived from oxygenated hemoglobin

A. Right (channel 1 and channel 2)

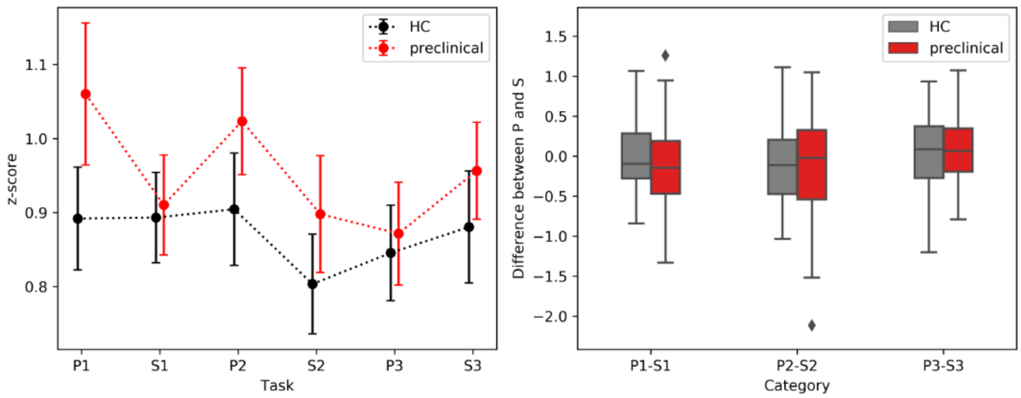

B. Inter 1 (channel 1 and channel 3)

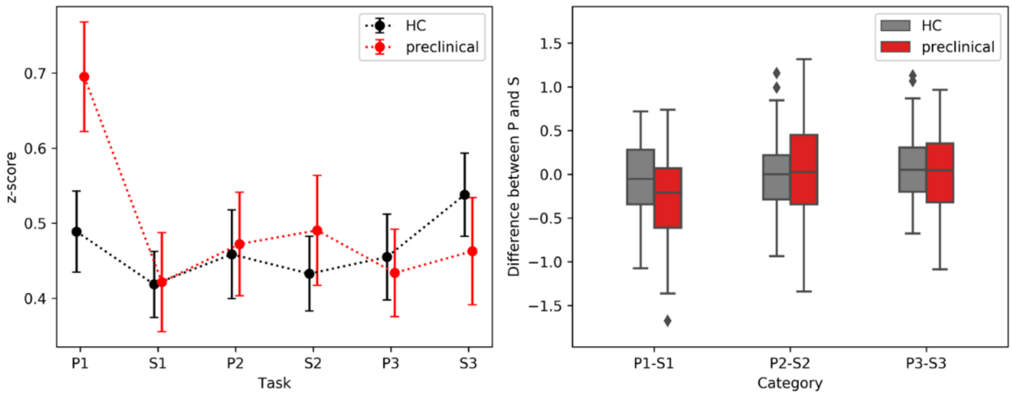

C. Inter 3 (channel 2 and channel 3)

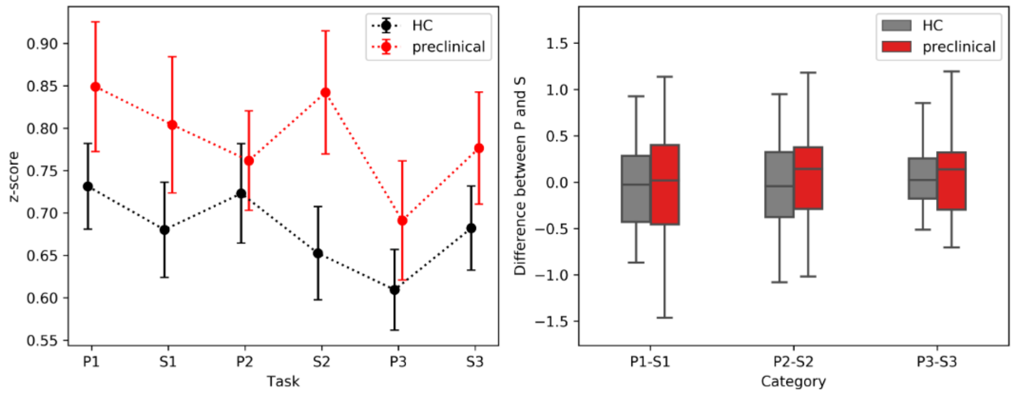

D. Inter 4 (channel 2 and channel 4)

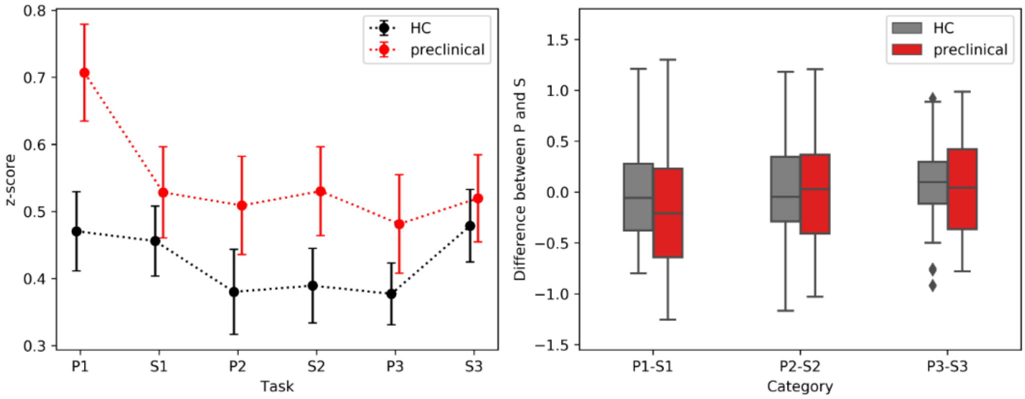

E. Left (channel 3 and channel 4)

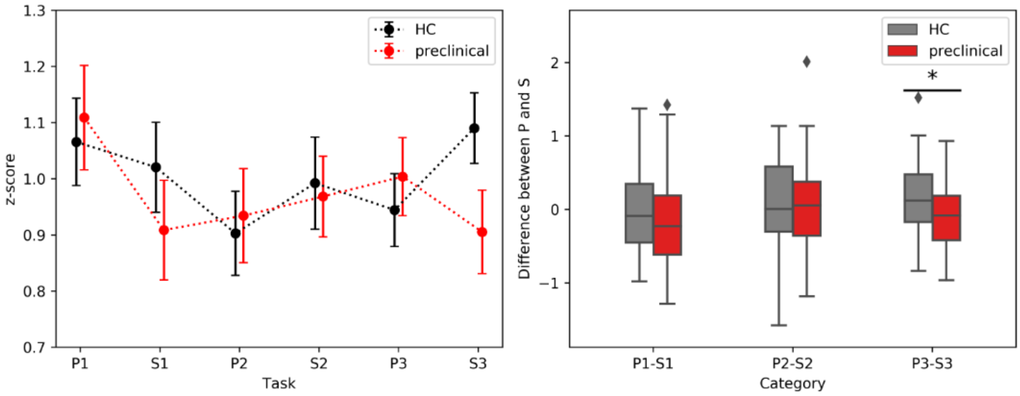

2. Functional connectivity derived from deoxygenated hemoglobin

A. Right (channel 1 and channel 2)

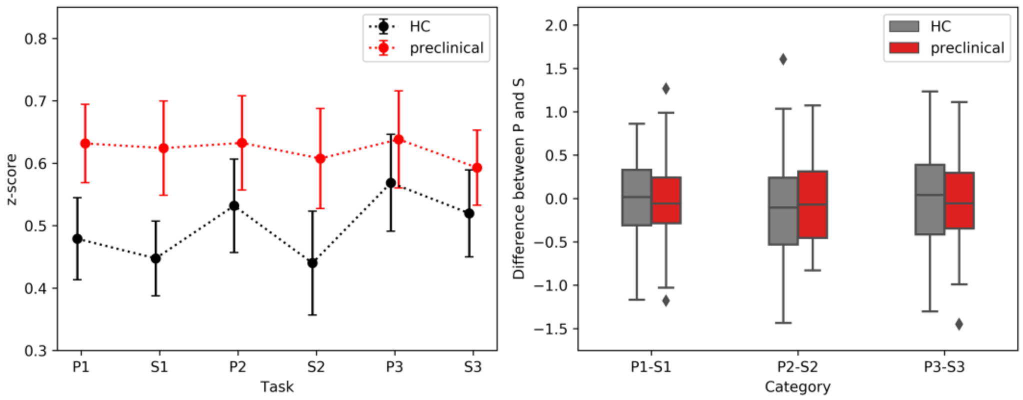

B. Inter 1 (channel 1 and channel 3)

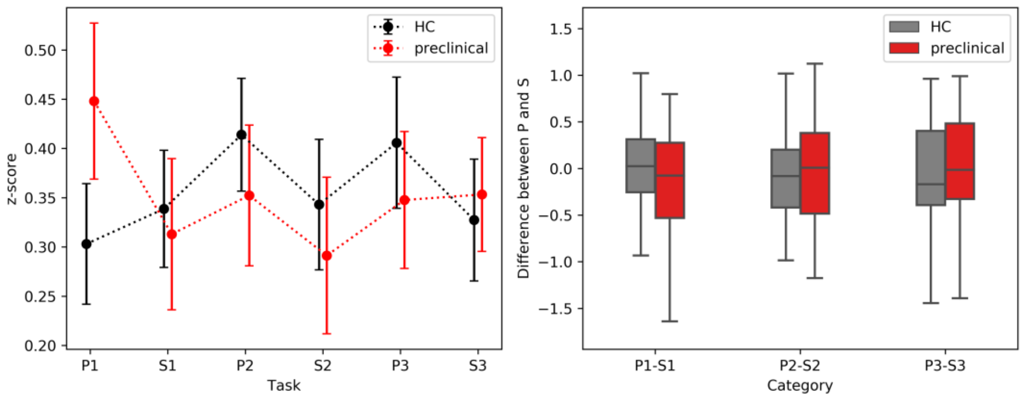

C. Inter 2 (channel 1 and channel 4)

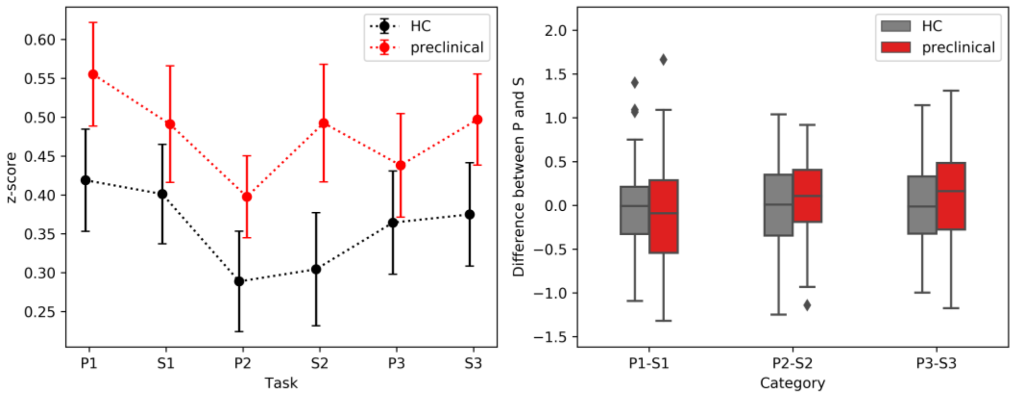

D. Inter 3 (channel 2 and channel 3)

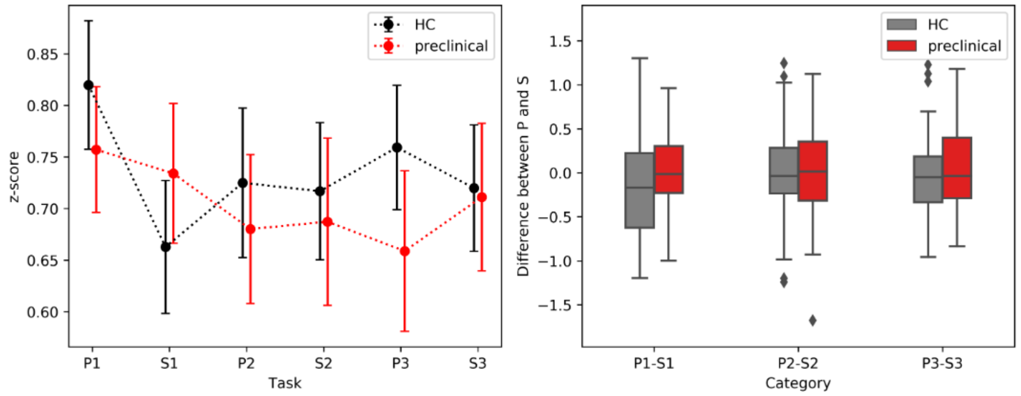

E. Inter 4 (channel 2 and channel 4)

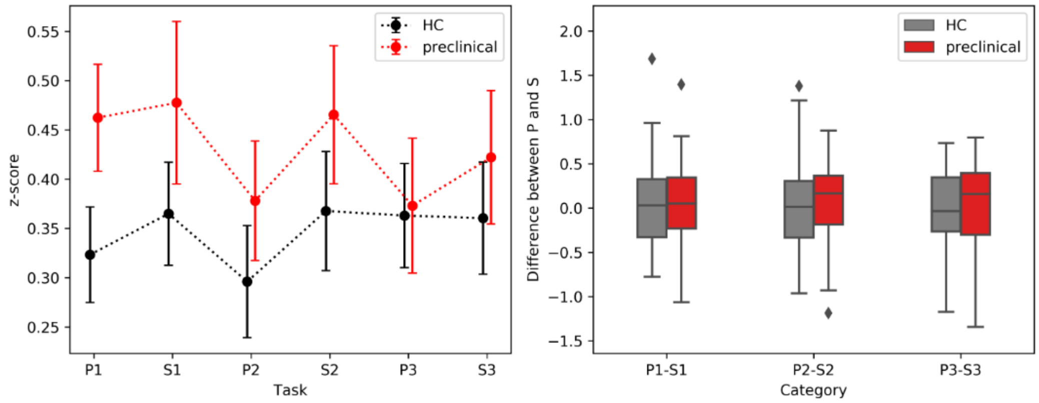

F. Left (channel 3 and channel 4)

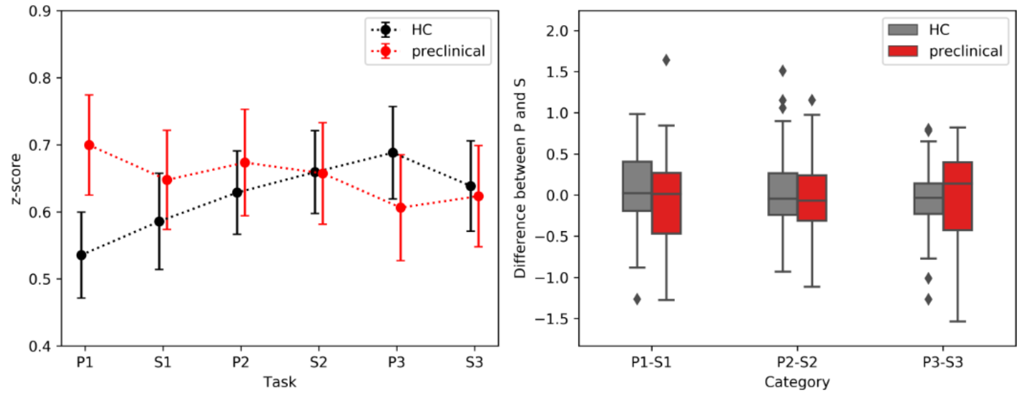

Figure S1. Functional connectivity difference between phonemic (P) and semantic (S) verbal fluency tasks. **(1)** Oxygenated hemoglobin and **(2)** Deoxygenated hemoglobin. Averages of inter2 FC during VFT on the left, and Inter FC difference between phonemic and semantic VFT on the right are displayed. The scatter plots on the left show the mean and standard error, while the boxplots on the right display the interquartile range, with whiskers extending to the lower and upper bound. The asterisk indicates FDR corrected $p < 0.05$.
